# BPIQ, a novel synthetic quinoline derivative, inhibits growth and induces mitochondrial apoptosis of lung cancer cells *in vitro* and *in zebrafish xenograft model*

**DOI:** 10.1186/s12885-015-1970-x

**Published:** 2015-12-16

**Authors:** Chien-Chih Chiu, Han-Lin Chou, Bing-Hung Chen, Kuo-Feng Chang, Chih-Hua Tseng, Yao Fong, Tzu-Fun Fu, Hsueh-Wei Chang, Chang-Yi Wu, Eing-Mei Tsai, Shinne-Ren Lin, Yeh-Long Chen

**Affiliations:** 1 Department of Biotechnology, Kaohsiung Medical University, Kaohsiung, 807 Taiwan; 2 Department of Medicinal and Applied Chemistry, Kaohsiung Medical University, Kaohsiung, 807 Taiwan; 3 Department of Biomedical Science and Environmental Biology, Kaohsiung Medical University; Cancer Center, Kaohsiung Medical University Hospital, Kaohsiung Medical University, Kaohsiung, 807 Taiwan; 4 School of Pharmacy, Kaohsiung Medical University, Kaohsiung, 807 Taiwan; 5 Department of Medical Laboratory Science and Biotechnology, School of Medicine, National Cheng Kung University, Tainan, 701 Taiwan; 6 Department of Thoracic Surgery, Chi-Mei Medical Center, Tainan, 710 Taiwan; 7 Department of Biological Sciences, National Sun Yat-Sen University, Kaohsiung, 804 Taiwan; 8 Translational Research Center, Cancer Center, Department of Medical Research, and Department of Obstetrics and Gynecology, Kaohsiung Medical University Hospital, Kaohsiung Medical University, Kaohsiung, 807 Taiwan; 9 Research Center for Environment Medicine, Kaohsiung Medical University, Kaohsiung, 807 Taiwan; 10 Institute of Biomedical Science, National Sun Yat-Sen University, Kaohsiung, Taiwan

**Keywords:** Indeno[1,2-*c*]quinolinequinoline, BPIQ, Lung cancer, Apoptosis, Polyploidy, Zebrafish xenograft

## Abstract

**Background:**

2,9-Bis[2-(pyrrolidin-1-yl)ethoxy]-6-{4-[2-(pyrrolidin-1-yl)ethoxy] phenyl}-11*H*-indeno[1,2-*c*]quinolin-11-one (BPIQ) is a derivative from 6-arylindeno[1,2-*c*]quinoline. Our previous study showed the anti-cancer potential of BPIQ compared to its two analogues topotecan and irinotecan. In the study, the aim is to investigate the potency and the mechanism of BPIQ against lung cancer cells.

**Methods:**

Both *in vitro* and zebrafish xenograft model were performed to examine the anti-lung cancer effect of BPIQ. Flow cytometer-based assays were performed for detecting apoptosis and cell cycle distribution. Western blot assay was used for detecting the changes of apoptotic and cell cycle-associated proteins. siRNA knockdown assay was performed for confirming the apoptotic role of Bim.

**Results:**

Both *in vitro* and zebrafish xenograft model demonstrated the anti-lung cancer effect of BPIQ. BPIQ-induced proliferative inhibition of H1299 cells was achieved through the induction of G_2_/M-phase arrest and apoptosis. The results of Western blot showed that BPIQ-induced G_2_/M-phase arrest was associated with a marked decrease in the protein levels of cyclin B and cyclin-dependent kinase 1 (CDK1). The up-regulation of pro-apoptotic Bad, Bim and down-regulation of pro-survival XIAP and survivin was observed following BPIQ treatment.

**Conclusions:**

BPIQ-induced anti-lung cancer is involved in mitochondrial apoptosis. BPIQ could be a promising anti-lung cancer drug for further applications.

**Electronic supplementary material:**

The online version of this article (doi:10.1186/s12885-015-1970-x) contains supplementary material, which is available to authorized users.

## Background

Lung cancer is one of the leading malignancies worldwide, and non-small cell lung cancer (NSCLC) accounts for at least 80 % of lung cancer [1]. Approximately one out of three patients with NSCLC has locally advanced disease that is surgically unavailable [2]. Nowadays, chemotherapeutic strategies for NSCLC therapy are constantly developed and improved [2–6]. However, the poor prognosis at an advanced stage of NSCLC and chemotherapeutic resistance contribute to the low survival rate of NSCLC patients [3].

**Fig. 1:**
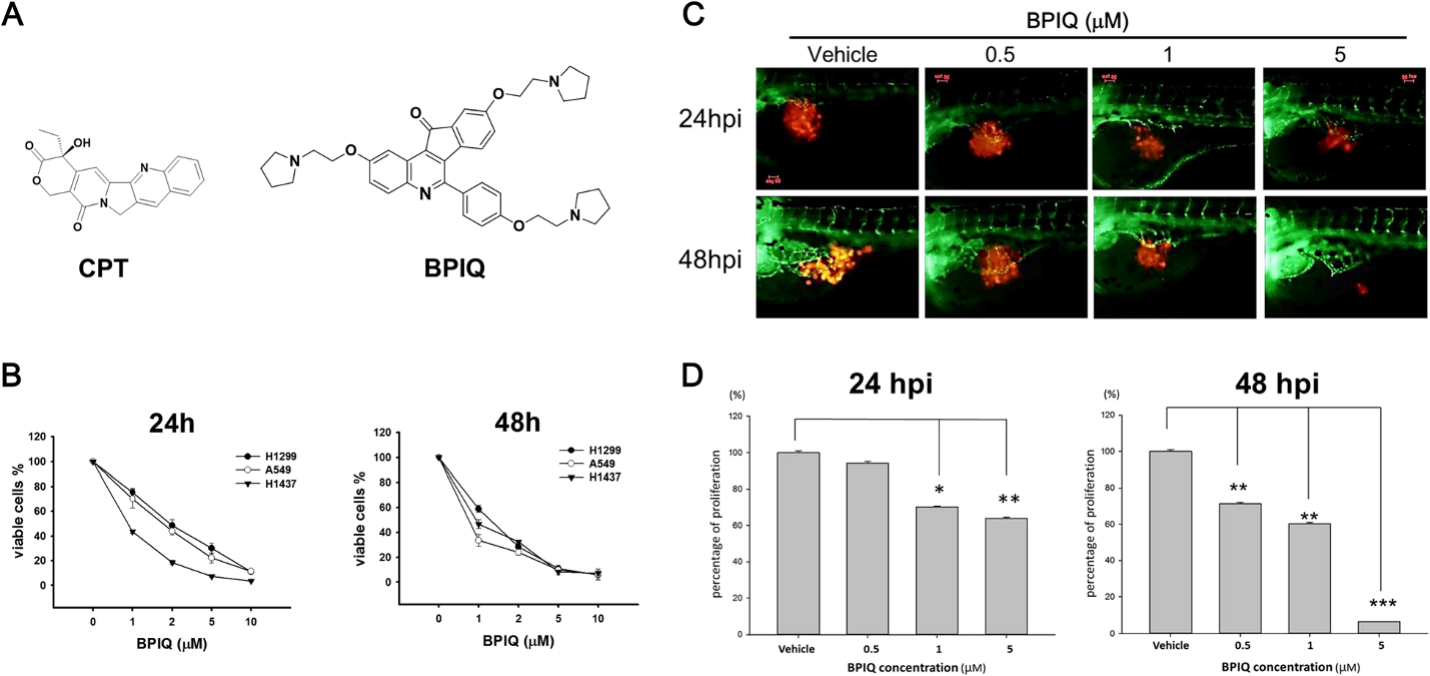
Effect of BPIQ on proliferation of NSCLC tumor cells. **a** The structures of CPT and BPIQ. **b** Three NSCLC H1299, A549 and H1437 cells were incubated with various concentrations of BPIQ for 24 and 48 h, respectively. The percentage of viable cells was calculated as a ratio of BPIQ- to DMSO-treated control cells. **c** The tumor volume in the zebrafish xenograft model. The intensity of red fluorescence is proportional to the xenograft tumor size. *N* = 20 embryos for each group. **d** The quantificative analysis of c. All data are presented as mean ± S.D. of three independent experiments. (*p < 0.05, **p < 0.005 and ***p < 0.001 against vehicle control, respectively)

Quinoline ring was found in a variety of biologically active compounds, which exert the anti-inflammation [7], anti-autoimmunity [8] and anti-cancer proliferative activities [7, 9–12]. The well-known quinoline derivative, camptothecin (CPT) is a pentacyclic quinoline isolated from the Chinese tree *Camptotheca acuminata*, which was reported to possess a potent cytotoxicity in a variety of cancers (Fig. 1a). CPT derivatives including irinotecan and topotecan are widely used as anti-cancer drugs [11]. However, the inherent chemical properties of CPT, including poor solubility and instability under physiological conditions, prevent its full clinical applications [13]. Accordingly, the quinoline derivatives are being developed to enhance the anti-tumor activity and reduce side effects [14, 15]. Subsequent introduction of hydrophilic side chains led to the discovery of topotecan and irinotecan which are currently used as anti-cancer drugs [11].

To overcome these aforementioned limitations and to improve the therapeutic potential of quinoline derivative, we synthesized a novel 2,9-bis[2-(pyrrolidin-1-yl)ethoxy]-6-{4-[2-(pyrrolidin-1-yl)ethoxy]phenyl}-11*H*-indeno[1,2-*c*]quinolin-11-one (BPIQ) [9, 11, 16]. Further, the previous study has demonstrated the anti-proliferation potential of BPIQ in several cancer cells, including NSCLC and hepatocellular carcinoma (HCC) tumor cells [9, 11]. Interestingly, the previous work showed that BPIQ exerts more strong toxicity towards lung cancer cell lines compared to other two BPIQ analogues, topotecan and irinotecan, which were used as anti-cancer drugs [17].

Despite the potent inhibitory effect of BPIQ on proliferation of NSCLC cancer cells, little is known about its underlying mechanism. To clarify the proliferation inhibition by BPIQ, cellular and molecular parameters pertaining to BPIQ-induced apoptosis were studied using three NSCLC tumor cells, H1299, H1437 and A549. In addition to the *in vitro* assays, we also performed the zebrafish xenograft to evaluate the anti-cancer potential of BPIQ, as well as its toxicity towards zebrafish larvae as the side-effect index.

## Methods

### Preparation of BPIQ and CPT

BPIQ (Fig. 1a) was synthesized as previously described [9, 11]. Camptothecin (CPT) was purchase from Sigma-Aldrich (St. Louis, MO, USA). Both BPIQ and CPT were dissolved in DMSO (less than 0.01 %) immediately prior to experiments.

### Reagents

The following compounds were obtained from Gibco BRL (Gaithersburg, MD, USA): DMEM medium, fetal bovine serum (FBS), trypan blue, penicillin G, and streptomycin. Dimethyl sulphoxide (DMSO), CPT, ribonuclease A (RNase A), and propidium iodide (PI) were purchased from Sigma-Aldrich. Antibodies against Bcl-2, XIAP, survivin, cytochrome *c*, Bax, Bad, PARP, and β-actin were obtained from Santa Cruz Biotechnology (Santa Cruz, CA, USA). Antibodies against cleaved caspase-3 and caspase-9 were purchased from Anaspec (San Jose, CA, USA). Anti-mouse and anti-rabbit IgG peroxidase-conjugated secondary antibodies were purchased from Pierce (Rockford, IL, USA). The anti-rabbit Rhodamine-conjugated antibody was purchased from Abcam (Cambridge, UK). Annexin V-Fluorescein isothiocyanate (FITC) staining kit was purchased from Strong Biotech (Taipei, Taiwan). The cationic cyanine dye, carbonyl cyanide 3-chlorophenylhydrazone (CCCP) included in DiOC_2_(3) assay kit was obtained from Invitrogen (Carlsbad, CA, USA).

### Cell culture

Human non-small cell lung cancer (NSCLC) cells H1299, H1437 and A549 were obtained from the American Type Culture Collection (ATCC; Manassas, VA, USA). All tested cells were maintained in DMEM: F-12/3:2 ratio and supplemented with 8 % FBS, 2 mM glutamine, and antibiotics (100 units/ml penicillin and 100 μg/ml streptomycin) at 37 °C in a humidified atmosphere of 5 % CO_2_. Before all assays performed in the study, all cells were tested to exclude the mycoplasma contamination using a PCR-based assay described by Wirth et al. [18].

### Proliferative inhibition assay

The cell proliferation rate and cell viability were determined by trypan blue dye exclusion assay combined with the Countess™ automated cell counter performed according to the manufacturer’s instruction (Invitrogen, Carlsbad, CA, USA). Briefly, 1 × 10^5^ cells were seeded and treated with DMSO as vehicle or the indicated concentrations of BPIQ for 24 h and 48 h. After incubation, cells were exposed to 0.2 % trypan blue and counted by Countess™ [19].

### Apoptosis assessment

To examine the apoptosis-inducing potential of BPIQ, Annexin-V/PI double staining was performed to detect the externalization of phosphatidylserine (PS). In brief, 5 × 10^5^ cells were seeded onto 100-mm petri dishes and treated with or without BPIQ for 24 h. Subsequently, cells were harvested and stained with Annexin V staining kit according to the manufacturer’s manual. Cells were analyzed by flow cytometry (FACS Calibur; Becton Dickinson, Mountain View, CA, USA) using WinMDI 2.9 software (written by Joseph Trotter, Scripps Research Institute, La Jolla, CA, USA).

### Mitochondria membrane potential (MMP) analysis

The changes of MMP were measured by flow cytometry using DiOC_2_(3) fluorescence dye following the manufacturer’s instructions. Cells were treated with 50 μM of carbonyl cyanide 3-chlorophenylhydrazone (CCCP) as a positive control. Data were analyzed using the CellQuest software (Becton Dickinson).

### Cytosolic extraction for Western blot

To determine whether BPIQ causes cytochrome *c* release, a protein extraction of cytosol fraction was conducted by the mitochondria protein extraction kit Bio-PMTF-60 (BioKit, Hsinchu, Taiwan). Briefly, a total of 5 × 10^6^ cells was harvested by centrifugation. Cell pellet was resuspended in reagent A and vortexed, then incubated on ice. The lysates were centrifuged to collect supernatants as cytosolic fraction and transfer to a fresh tube and added reagent B to each precipitation part, vortex for homogeneous solution and centrifugation. Finally, the cytosolic fractions were further analyzed by Western blotting.

### Western blot analysis

Western blotting was performed as described previously [20]. Briefly, cells were harvested and lysed. Lysates were centrifuged, and the protein concentration was determined. Equal amounts of protein were separated by SDS-polyacrylamide gel electrophoresis (SDS-PAGE) and then electrotransferred. The membrane was blocked with 5 % non-fat milk, followed by incubation with primary and secondary antibodies against specific proteins. The signals were detected using enhanced chemiluminescence (ECL) detection kit (Amersham Piscataway, NJ, USA).

### Immunofluorescence assay

To determine whether BPIQ causes the release of cytochrome c, the immunofluorescence assay was conducted according to a previous study with minor modifications [1]. In brief, H1299 and A549 cells were grown on 12-mm glass coverslips (Marienfeld Laboratory, Lauda-Königshofen, Germany) respectively. Cells treated with BPIQ were attached using 37 % nitric acid (Sigma-Aldrich), fixed with 4 % paraformaldehyde and permeabilized with 0.5 % Tween-20. Cells were incubated overnight at 4 °C with the primary antibody against cytochrome c (#sc13156, Santa Cruz Biotechnology), washed with 1 % Bovine serum albumin (BSA), the incubated with Alexa Fluor 555–conjugated goat anti–mouse immunoglobulin G (#A21422, Molecular Probes, Invitrogen, Carlsbad, CA). The slides were mounted in fluorescent mounting medium Vectashield H-1000 (Vector Laboratories, Burlingame, CA, USA).

### siRNA knockdown assay

Bim siRNA (Hs_BCL2L11) was purchased from Bertec, Taiwan. Bim siRNA or a scrambled sequence control was transfected into H1299 cells using the transfection reagent RNAi Lipofectamine according to the manufacture instruction (Invitrogen, Life Technologies, Carlsbad, CA, USA). After 24 h of transfection, the medium was refreshed, and the cells were incubated at 37 °C with a humidified atmosphere of 5 % CO_2_ for an additional 24 h [1].

### Zebrafish xenograft assay

The zebrafish (Danio rerio) Tg(fli1:EGFP) were obtained from Taiwan

Zebrafish Core Facility at Academia Sinica (TZCAS, Taipei, Taiwan). The care and maintenance of zebrafish were handled in compliance with the animal care regulations and standard protocols of the animal center (Kaohsiung Medical University Hospital, Kaohsiung, Taiwan) for zebrafish adults and larvae). Zebrafish were kept at 28.5 °C in aquaria with day/night light cycles (10 h dark *vs*. 14 h light periods).

### Zebrafish xenograft assay

The zebrafish xenograft assay was used for confirming the inihibitory effect of BPIQ on proliferation of lung cancer cells. The use of zebrafish complied with the principles of 3Rs (Reduction, Replacement and Refinement) and the approval protocol (IACUC Approval No. KMU-IACUC-102033) by Institutional Animal Care and Use Committee (IACUC) of Kaohsiung Medical University Hospital, Kaohsiung, Taiwan We transfected a red fluorescent protein from pDsRed-Express-C1 (Clontech, Mountain View, CA, USA) into human lung tumor cells for tracking in the zebrafish xenograft model with a fluorescence microscopy. The procedure was performed according to a previous study with minor modifications [20]. Briefly, 48 h post-fertilization (hpf) zebrafish embryos were anesthetized with 0.01 % of tricaine and transplanted with about 50 lung cancer cells per embryo. Cells then were resuspended in Hanks balanced salt solution and injected into the yolk sac of the embryos. The embryos were incubated in water at indicated concentrations of BPIQ for 24 and 48 h post-injection (hpi), respectively. Afterwards, photographs of embryos were taken by an inverted microscope (Nikon Eclipse TE2000-U, Tokyo, Japan).

### Statistical analysis

Differences between BPIQ- and DMSO- (as vehicle control) treated cells were analyzed in at least triplicate experiments. The significance of the differences was analyzed by one-way analysis of variance (ANOVA), with *p* < 0.05 considered significantly.

## Results

### BPIQ exerts the anti-lung cancer potential both in vitro and in vivo

To examine the effect of BPIQ on cell growth, three NSCLC tumor cell lines H1299 (null p*53*), A549 (wild type p*53*) and H1437 (mutant p53-R267P) were treated with increasing concentrations of BPIQ for 24 h and 48 h. Cell survival was assessed by trypan blue exclusion combined with an automated cell counter. As shown in Fig. 1b, significant inhibition of proliferation was detected at 1, 2, 5 and 10 μM BPIQ in both dose- and time-dependent manners. Both the IC_50_ of BPIQ and CPT at 24 h and 48 h for three NSCLC cell lines are shown for comparison in Tables 1 and 2 (The proliferation inhibition curve for CPT is shown in the Additional file 1: Figure S1). We further examined whether BPIQ inhibits the growth of NSCLC cells *in vivo*. H1299 cells, the most invasive among three tested NSCLC cells, were implanted into the yolk sac of zebrafish larvae for 72 h followed by incubating larvae with different BPIQ concentrations for the indicated times. Consistently, the zebrafish xenograft assay further confirmed the anti-lung cancer potential of BPIQ (Fig. 1c and d) in that the observed tumor sizes, as indicated by the intensity of red fluorescence, were reversely proportional to BPIQ concentrations in zebrafish larvae.

**Table 1:** The comparison of CPT and BPIQ on anti-lung cancer activity. a IC_50_ values for BPIQ-treated NSCLC cells

Cell line (IC_50_ of BPIQ,μM)			
Time	H1299	H1437	A549
24 h	1.96	0.89	1.78
48 h	1.30	0.93	0.75

**Table 2:** The comparison of CPT and BPIQ on anti-lung cancer activity. b IC_50_ values for CPT-treated NSCLC cells

Cell line (IC_50_ of CPT,μM)			
Time	H1299	H1437	A549
24 h	2.73	N.D.	3.20
48 h	1.60	N.D.	1.55

*N.D*. Not determined

### BPIQ causes G_2_/M arrest and aberrant polyploidy in H1299 cells

**Fig. 2:**
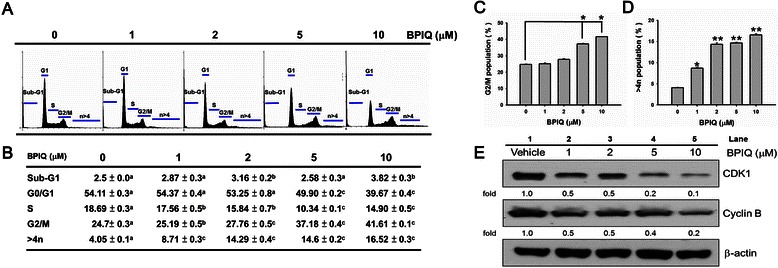
BPIQ induced an accumulated G_2_/M population and aberrant polyploidy in H1299 cells. Cells were treated with the indicated doses (vehicle control, 1, 2, 5, and 10 μM) of BPIQ for 24 h, respectively. **a** The accumulation of the G_2_/M population in BPIQ-treated H1299 cells and vehicle controls at 24 h. **b** The quantification analysis of cell cycle distribution. Different letter notations indicate the statistical significance between BPIQ treatment and vehicle (a *vs*. b and a *vs*. c indicate the *p* < 0.005 and *p* < 0.001, respectively.). **c** Analysis of G_2_/M population. **d** Analysis of polyploidy. Data are presented as means ± S.D. (*n* = 3). Different letter notations indicate the statistical significance between drug treatment and vehicle (*p < 0.05 and **p < 0.001 respectively). **e** Western blot analysis demonstrating BPIQ-induced down-regulation of CDK1 and cyclin B protein levels. β-actin was measured as an internal control

As shown in Fig. 2a and b, the G_2_/M population percentiles of H1299 cells treated with vehicle control and various BPIQ concentrations (1, 2, 5 and 10 μM) were 24.7 ± 0.3, 25.19 ± 0.5, 27.76 ± 0.5, 37.18 ± 0.4, and 41.61 ± 0.1 (*n* = 3), respectively. BPIQ induced accumulation of G_2_/M population of H1299 lung cancer cells increased in a dose dependent manner (Fig. 2c). Additionally, the BPIQ-induced polyploidy population (>4 N DNA) was slightly increased at a dose of 1 μM (*p* < 0.05) compared to untreated cells and became more significantly increased at the higher doses of 2, 5 and 10 μM (*p* < 0.0001) (Fig. 2d). Furthermore, the decreased protein levels of G_2_/M effectors cdk1 and cyclin B were also observed in a dose-dependent manner (Fig. 2e).

### Apoptosis was triggered by BPIQ in H1299 cells efficiently

**Fig. 3:**
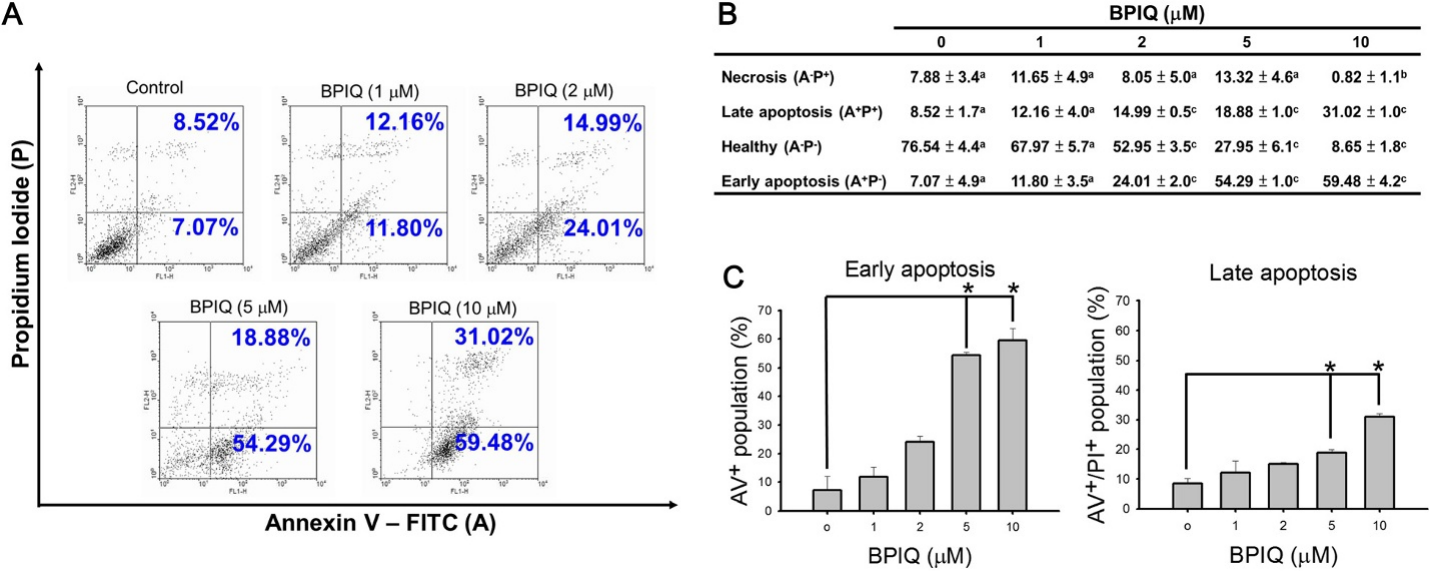
BPIQ induced apoptosis of H1299 cells. **a** Cells cultured with different concentrations of BPIQ for 24 h were stained with Annexin V/PI to detect externalization of PS from cell membrane. **b** Quantitative analysis of Annexin V staining. **c** Quantitative analysis of apoptotic cells. Different letter notations indicate the statistical significance between BPIQ treatment and vehicle (a *vs*. b and a *vs*. c indicate the *p* < 0.005 and *p* < 0.001, respectively.)

To determine whether BPIQ inhibits cell survival by inducing apoptosis, the flow cytometry based- Annexin V/PI dual staining was performed. H1299 cells cultured with different concentrations of BPIQ for 24 h were stained with Annexin V/PI to detect the externalization of PS from the cell membrane. In this assay, Annexin V^−^/PI^−^ cells were considered healthy, Annexin V^−^/PI^+^ cells were considered necrotic, Annexin V^+^/PI^−^ cells were considered early apoptotic, and Annexin V^+^/PI^+^ cells were considered late apoptotic. After treatment with vehicle control or 1, 2, 5 and 10 μM of BPIQ for 24 h, the cells displayed early- and late-stage of apoptosis as shown in Fig. 3. BPIQ caused a dose-dependent increase in the percentage of both early and late apoptotic cells (Fig. 3a and b), and the apoptosis-promoting capacity of BPIQ was significant at either 5 or 10 μM (Fig. 3c). These results showed that BPIQ efficiently induced apoptosis of H1299, suggesting that BPIQ inhibited proliferation of H1299 cells through induction of apoptosis.

### BPIQ induces the disruption of MMP and mitochondrial-mediated apoptosis

**Fig. 4:**
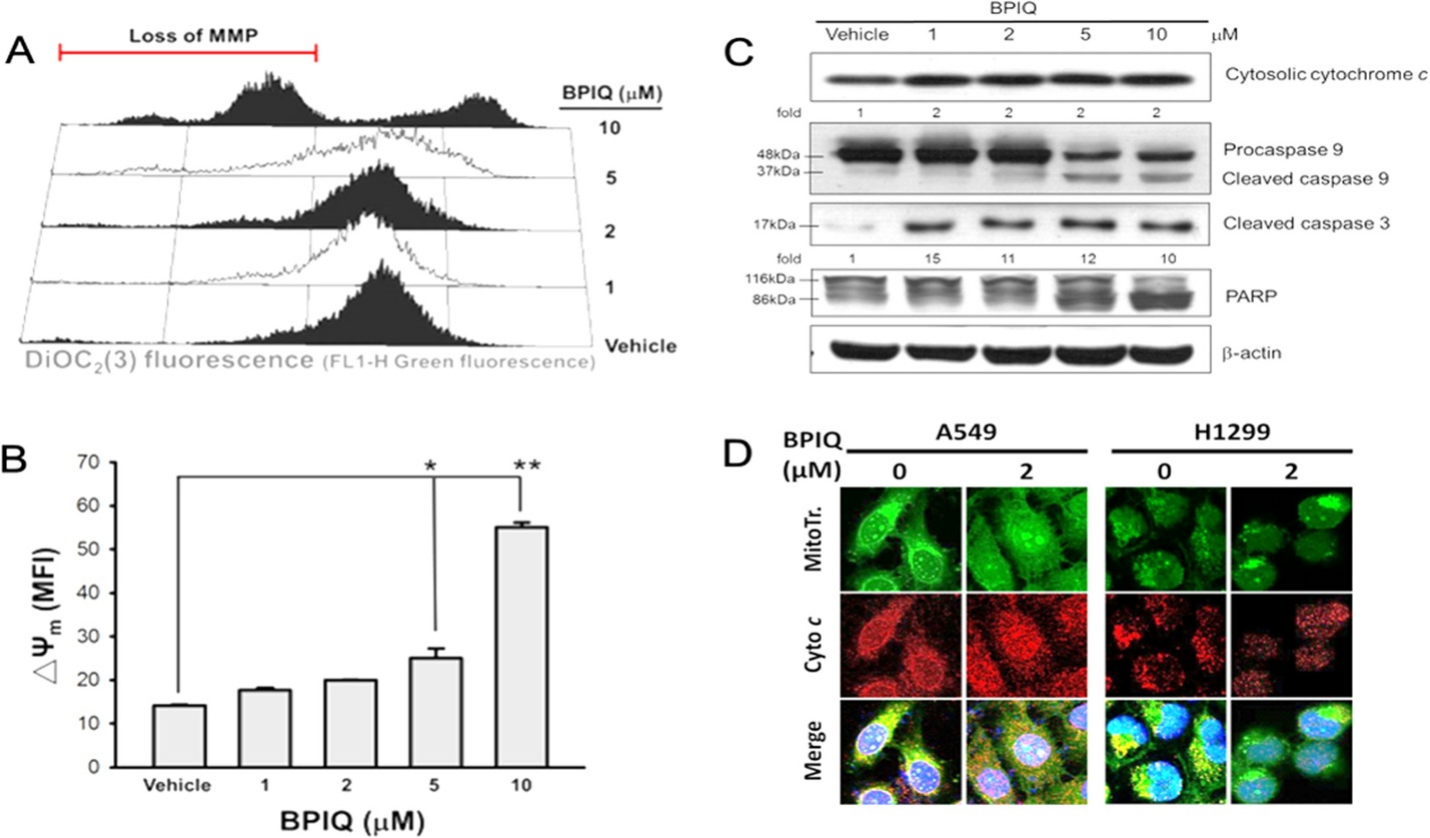
Loss of MMP and caspase activation by BPIQ. **a** H1299 cells were exposed to media containing the indicated concentrations of BPIQ or vehicle control for 24 h, stained with DiOC_2_(3), then analyzed for changes in their fluorescent profile by flow cytometry. **b** Quantitative analysis. Data are presented as means ± S.D. Histograms represent one of three independent experiments. *p < 0.05 and **p < 0.001 against vehicle control, respectively. **c** Western blot analysis demonstrating BPIQ-induced cytochrome c release and cleavage of caspase-9 and −3, as well as PARP. β-actin was measured as an internal control. **d** The distribution of cytochrome *c* in the cytosol of two NSCLC cell lines A549 and H1299 following 2 μM BPIQ treatment. ▪ mitochondria; ▪ cytochrome *c*; ▪ DAPI; ▪ co-localization of mitochondria and cytochrome *c*. Magnification 200 x

As depicted in Fig. 4a, BPIQ induced disruption of MMP. Furthermore, Fig. 4b showed the quantitative values. The MMP changes (Δψm) induced by various BPIQ concentrations were 14.14 ± 0.22 (vehicle control), 17.69 ± 0.58 (1 μM), 19.92 ± 0.13 (2 μM), 25.06 ± 2.16 (5 μM), 55.04 ± 1.09 (10 μM), respectively. Additionally, the MMP change in cells treated with CCCP (50 μM) as positive control was 36.72 ± 0.7. These results suggested that BPIQ potentially triggers the disruption of MMP, the hallmark of mitochondrial mediated apoptosis in a dose-dependent manner. Furthermore, other major hallmarks of apoptosis, including the release of cytochrome *c*, cleaved caspase-9 and −3, as well as cleaved form of PARP were detected at higher BPIQ concentrations used (Fig. 4c, lanes 4 and 5). Likewise, the immunofluorescence assay showed that the BPIQ causes the redistribution of cytochrome c into the cytosol of H1299 cells (Fig. 4d. The yellow fluorescence indicates the colocalization of cytochrome *c* and mitochondria, and the red fluorescence indicates the distribution of cytochrome *c*).

### BPIQ disturbs the balance of pro-survival and pro-apoptosis Bcl-2 family proteins

**Fig. 5:**
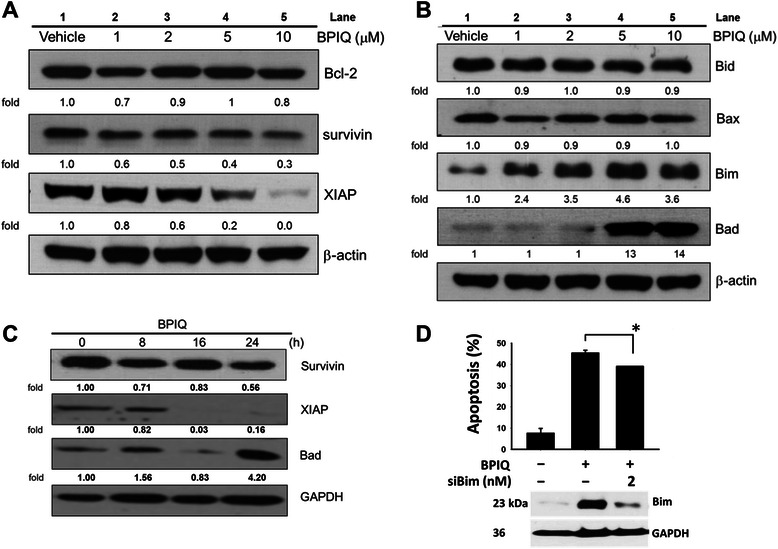
The effects of BPIQ on modulation of Bcl-2 family members in H1299 cells. Cells were subjected to treatment with vehicle control or the indicated doses of BPIQ. **a** Western blot showed the significantly decreased levels of IAP factors survivin and XIAP. **b** Western blot showed the increase in pro-apoptotic Bid, Bad and Bim protein levels. **c** Western blot showed BPIQ causes the changes of IAP factors and pro-apoptotic Bad in a time dependent-manner. β-actin was measured as an internal control. Each blot is representative of three independent experiments. **d** The effect of Bim knockdown on BPIQ-induced apoptosis of H1299 cells determined using a cytometry-based annexin v staining assay. *p < 0.05 for scramble siRNA versus. Bim siRNA

To examine the effects of BPIQ treatment on protein levels involved in apoptosis, H1299 cells were treated with various concentrations of BPIQ for 24 h before cell lysates were harvested and subjected to Western blot analyses. As shown in Fig. 5a, BPIQ significantly decreased the levels of pro-survival proteins survivin and XIAP, whereas no significant changes of Bcl-2 protein were observed. On the contrary, the levels of two pro-apoptotic proteins, Bim and Bad, were dramatically increased following BPIQ treatment in a dose-dependent manner (Fig. 5b). Figure 5c showed the protein level changes of survivin, XIAP and Bad in BPIQ-treated H1299 cells in a time-course manner. Furthermore, the knockdown assay confirmed the pro-apoptotic role of Bim in BPIQ-induced apoptosis of H1299 cells (Fig. 5d).

## Discussion

Due to the poor prognosis in advanced human NSCLC tumors, screening compounds which selectively exhibit apoptosis-inducing capability is the urgent goal for NSCLC chemotherapy. Our previous study showed that the synthetic quinoline derivative BPIQ is an anti-growth agent against lung cancer and liver tumor cells [9, 11]. The values of 50 % growth inhibition (GI_50_) of the topotecan- and irinotecan-treated A549 lung cancer cells at 24 h were 5.98 ± 0.26 μM and > 10 μM respectively. Likewise, both the GI_50_ values of the topotecan- and irinotecan-treated H1299, an invasive lung cancer cells at 24 h were higher than >10 μM. In comparison of the CPT analogues, our previous results showed that BPIQ exhibits a significantly cytotoxicity against both NSCLC cells lines at 24 h (GI_50_, 0.67 ± 0.01 μM and 0.37 ± 0.07 μM, respectively) (compound 15 as BPIQ in Table 1.) [9, 11].

To evaluate the efficacy of CPT and BPIQ on suppressing growth of lung cancer cells, the proliferation assay was also conducted. The results showed that IC_50_ of CPT for H1299 cells was 2.73 (24 h) and 1.6 μM (48 h), respectively, and the IC_50_ of CPT for A549 cells was 3.20 (24 h) and 1.55 μM (48 h), respectively (Additional file 1: Figure S1). These results suggest that the inhibitory efficacy of BPIQ is moderately better than CPT. The safety of BPIQ for clinical applications should be worthy for evaluating in our further *in vivo* study.

Accordingly, in this study, we further demonstrated the anti-proliferative effect of BPIQ on human NSCLC cells, including H1299, H1435, as well as H1437. The results confirmed that BPIQ effectively inhibited the proliferation of all tested NSCLC tumor cells (Fig. 1b and c).

Because of the advantages of small size, embryonic transparency and rapid development, zebrafish (*Danio rerio*) is widely used as an ideal model organism [21, 22]. Furthermore, the physiological responses in zebrafish to tested compounds can be comparable to those in mammalian models [22]. Recently, zebrafish xenograft assay is becoming a useful tool for investigating and tracking human cancer cells in zebrafish larvae, such as invasion, tumor proliferation [23] and angiogenesis [24]. The transparency of zebrafish embryos and larvae makes the xenograft assay to be readily performed for observing tumor proliferation and interactions between cancer cells and the microenvironment in zebrafish. Importantly, the zebrafish xenograft assay can evaluate both the activity and side effect of a tested compound [9, 11]. Therefore, to further validate the anti-lung cancer effects of BPIQ, we conducted the zebrafish xenograft assay. Consistently, the results of zebrafish xenograft assay showed the inhibitory effect of BPIQ on lung cancer cells. However, we also found that the highest dose (5 μM) of BPIQ caused a significantly toxicity towards zebrafish larvae (data not shown), suggesting that the dose usage of BPIQ should be more careful when further applied for lung cancer chemotherapeutics. Nevertheless, these observations indicate that BPIQ may have the potential for lung cancer treatment in the future.

To uncover the molecular mechanism of BPIQ-mediated inhibition on NSCLC cells proliferation, we examined the effect of BPIQ on cell cycle distribution of H1299 cells. The cell cycle analysis showed that BPIQ induced a moderate accumulation of G_2_/M population (Fig. 2a), which was accompanied by polyploidy (>4n) (Fig. 2b and d). Recent studies showed that certain anticancer drugs exert their effects through destabilizing the genome and causing aberrant polyploidy. For example, the aurora B kinase inhibitor ADZ1522 causes an increased proportion of polyploidy cells [25] and apoptotic cell death of colorectal cancer cells SW620 [13]. Moreover, doxorubicin could induce genome instability and polyploidy and cause the senescence of HCT116 colon cancer cells [26]. Consistently, we found that BPIQ caused significant accumulation of G_2_/M population and the aberrant polyploidy. Furthermore, the CDK1-cyclin B1 complex regulates entry of cell cycle into mitosis, and the decreased levels or loss of activities of cyclin B1 and CDK1 causes the G_2_/M arrest and may promote apoptotic cell death [27, 28]. Our current study showed that protein levels of CDK1 and cyclin B1 were dramatically decreased by BPIQ treatments. These observations suggest that BPIQ-induced growth inhibition is associated with G_2_/M arrest and the aberration of polyploidy.

Annexin V/PI double staining showed that BPIQ significantly induced apoptotic cell death, and caused proteolytic activation of caspase-3 and −9, as well as proteolytic inactivation of PARP (Fig. 3). Since BPIQ induced the disturbance of MMP and the release of cytochrome *c*, we suggest that BPIQ-induced apoptosis of H1299 cells is mitochondria-mediated (Fig. 4a and b).

Numerous studies suggest that mitochondria play an important role in cell survival and cytochrome c-mediated apoptosis by modulating the balance of pro-apoptotic and anti-apoptotic Bcl-2 family proteins [29–31]. For example, anthocyanin, a member of the flavonoid family, induces apoptosis of leukemia U937 cells by down-regulating Bcl-2 expression [32]. On the contrary, up-regulation of pro-apoptotic protein Bim was observed in glucocorticoid-induced apoptosis of acute lymphoblastic leukemia CEM cells [33]; and matrine, a sophora alkaloid, induced cell death of colorectal cancer through up-regulating bad expression [34]. Additionally, the inhibitors of apoptosis proteins (IAPs) also play important roles in negative regulation of apoptosis [35, 36]. Our result showed that BPIQ treatment increased protein levels of pro-apoptotic Bim and Bad, and this may disturb the balance of Bcl-2 family proteins. Additionally, dramatically decreased levels of two IAP proteins, survivin and XIAP, were detected (Fig. 5a and b). These observations are consistent with previous studies that increased levels of pro-apoptotic proteins induce cellular apoptosis [37, 38].

To further determine whether BPIQ could disturb the balance between pro-survival Bcl-2 protein and the endogenous inhibitors such as XIAP and survivin factor and pro-apoptotic Bcl-2 proteins in a time-dependent manner, expression levels of several Bcl-2 family proteins were determined using a time-course experiment. As shown in Fig. 5c, the results of Western blot showed that the levels of pro-survival IAP proteins, including XIAP and survivin, were decreased at 16 and 24 hr, respectively, following BPIQ treatment. On the contrary, the protein level of Bad was dramatically increased at 24 h, suggesting BPIQ-induced a disturbance of anti-apoptosis and pro-apoptosis Bcl-2 family in a time-dependent manner. To validate whether the up-regulation of Bim is involved in BPIQ-induced apoptosis, we also performed the knockdown experiments. The results of Annexin V-assay showed that Bim knockdown protects H1299 cells from undergoing apoptosis induced by BPIQ (*p > 0.05). Although apoptosis-inducing dose (5 μM) of BPIQ causes a significant increased level of Bim, the Annexin V staining assay showed that blockage of Bim partially rescues H1299 cells from BPIQ-induced apoptosis. The may due to the efficiency of siRNA transfection, and we suggest that Bim knockout may improve the rescues of BPIQ-induced apoptosis. Nevertheless, our results demonstrate the involvement of Bim in mediating BPIQ-induced apoptosis of H1299 cells.

The induction of apoptosis can initiate through two distinct pathways: the intrinsic apoptotic and the extrinsic apoptotic pathways [39]. Therefore, we also examined whether the extrinsic apoptotic pathway (death receptor-pathway) is also activated by BPIQ treatment, and the preliminary results of Western blot showed that no significant changes of caspase-8, a key caspase of the extrinsic apoptotic pathway was observed (data not shown). However, we still can not exclude the possibility that other extrinsic death pathway, such as caspase-10, coordinately involves BPIQ-induced apoptosis. Accordingly, our present results suggest that BPIQ induces apoptosis of lung cancer H1299 cells through mitochondria-mediated, an intrinsic pathway.

## Conclusion

**Fig. 6:**
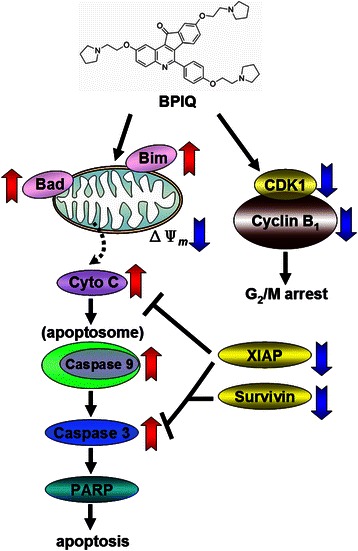
Schematic diagram of BPIQ-induced cell cycle arrest and apoptosis in H1299 cells. BPIQ causes G_2_/M arrest and aberrant polyploidy by decreasing cyclin B and CDK1 protein levels. Additionally, the down-regulation of pro-survival XIAP and survivin proteins, and the up-regulation of pro-apoptotic Bim and Bad result in a disruption of MMP potential. Subsequently, this triggers a mitochondria-mediated caspase cascade, then in turn induces apoptosis of H1299 cells

Our present work suggests that BPIQ exerts the anti-lung cancer cells both *in vitro* and *in vivo*. BPIQ-induced apoptosis was accompanied by perturbing the balance of pro- and anti-apoptotic Bcl-2 proteins by down-regulating levels of survivin and the IAP protein XIAP, and up-regulating levels of two pro-apoptotic proteins, Bim and Bad (Fig. 6). Our study sheds a light on the mechanism of BPIQ-based NSCLC chemotherapy.

## Additional file

Additional file 1: Figure S1. The effect of CPT on cell proliferation of lung cancer cells. (TIFF 151 kb)

## Abbreviations

BPIQ: 2,9-Bis[2-(pyrrolidin-1-yl)ethoxy]-6-{4-[2-(pyrrolidin-1-yl)ethoxy] phenyl}-11H-indeno[1,2-c]quinolin-11-one
CCCP: Carbonyl cyanide 3-chlorophenylhydrazone
CPT: Camptothecin
CDK1: Cyclin-dependent kinase 1
DMSO: Dimethyl sulphoxide
ECL: Enhanced chemiluminescence
hpf: Post-fertilization
hpi: Hour post-injection
MMP: Mitochondria membrane potential
PS: Phosphatidylserine
RNase A: Ribonuclease A
