# Supplementary figures and images for: BPIQ, a novel synthetic quinoline derivative, inhibits growth and induces mitochondrial apoptosis of lung cancer cells in vitro and in zebrafish xenograft model
Source: BMC Cancer. 2015 Dec 16;15:962. doi: 10.1186/s12885-015-1970-x (PMC4682281; doi:10.1186/s12885-015-1970-x)

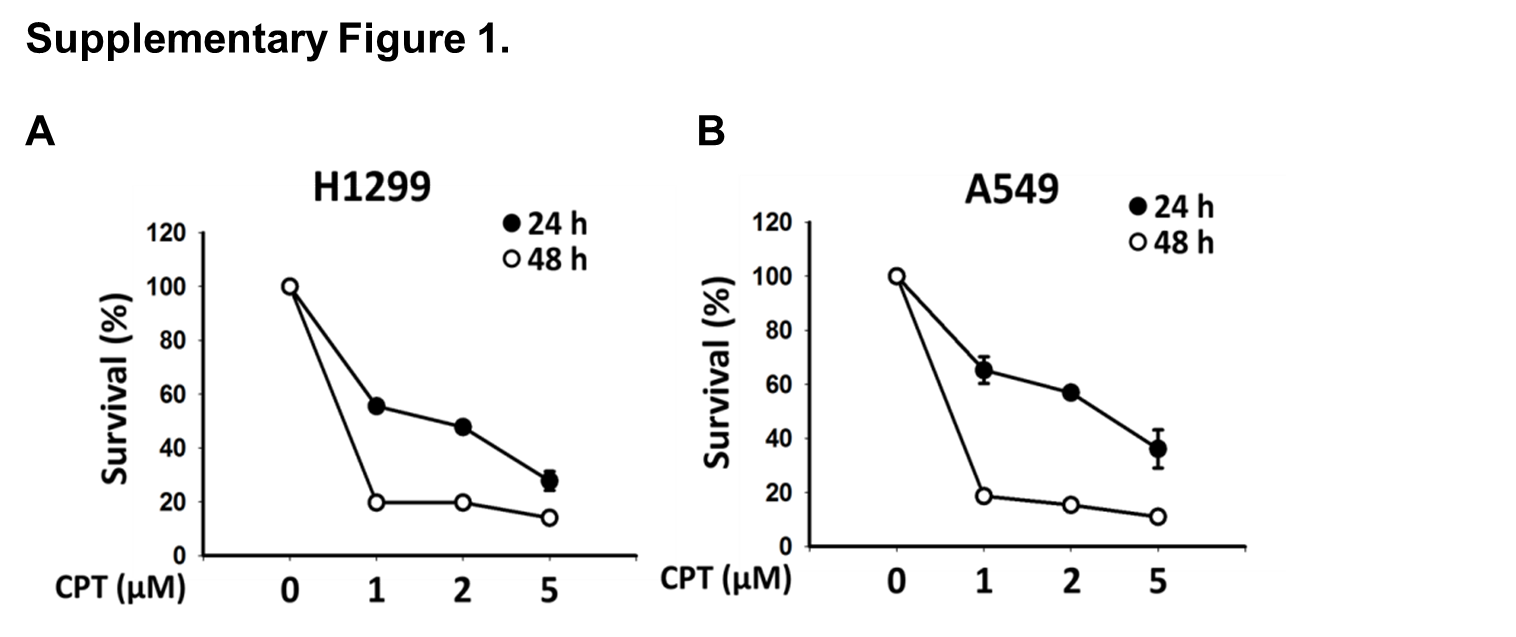

Supplement: Additional file 1: Figure S1. — The effect of CPT on cell proliferation of lung cancer cells. (TIFF 151 kb) [file 12885_2015_1970_MOESM1_ESM.tiff]
